# Identification of a Single Strand Origin of Replication in the Integrative and Conjugative Element ICE*Bs1* of *Bacillus subtilis*

**DOI:** 10.1371/journal.pgen.1005556

**Published:** 2015-10-06

**Authors:** Laurel D. Wright, Christopher M. Johnson, Alan D. Grossman

**Affiliations:** Department of Biology, Massachusetts Institute of Technology, Cambridge, Massachusetts, United States of America; University of Geneva Medical School, SWITZERLAND

## Abstract

We identified a functional single strand origin of replication (*sso*) in the integrative and conjugative element ICE*Bs1* of *Bacillus subtilis*. Integrative and conjugative elements (ICEs, also known as conjugative transposons) are DNA elements typically found integrated into a bacterial chromosome where they are transmitted to daughter cells by chromosomal replication and cell division. Under certain conditions, ICEs become activated and excise from the host chromosome and can transfer to neighboring cells via the element-encoded conjugation machinery. Activated ICE*Bs1* undergoes autonomous rolling circle replication that is needed for the maintenance of the excised element in growing and dividing cells. Rolling circle replication, used by many plasmids and phages, generates single-stranded DNA (ssDNA). In many cases, the presence of an *sso* enhances the conversion of the ssDNA to double-stranded DNA (dsDNA) by enabling priming of synthesis of the second DNA strand. We initially identified *sso1* in ICE*Bs1* based on sequence similarity to the *sso* of an RCR plasmid. Several functional assays confirmed Sso activity. Genetic analyses indicated that ICE*Bs1* uses *sso1* and at least one other region for second strand DNA synthesis. We found that Sso activity was important for two key aspects of the ICE*Bs1* lifecycle: 1) maintenance of the plasmid form of ICE*Bs1* in cells after excision from the chromosome, and 2) stable acquisition of ICE*Bs1* following transfer to a new host. We identified sequences similar to known plasmid *sso*'s in several other ICEs. Together, our results indicate that many other ICEs contain at least one single strand origin of replication, that these ICEs likely undergo autonomous replication, and that replication contributes to the stability and spread of these elements.

## Author Summary

Mobile genetic elements facilitate movement of genes, including those conferring antibiotic resistance and other traits, between bacteria. Integrative and conjugative elements (ICEs) are a large family of mobile genetic elements that are typically found integrated in the chromosome of their host bacterium. Under certain conditions (e.g., DNA damage, high cell density, stationary phase) an ICE excises from the host chromosome to form a circle. A linear single strand of ICE DNA can be transferred to an appropriate recipient through the ICE-encoded conjugation machinery. In addition, following excision from the chromosome, at least some (perhaps most) ICEs undergo autonomous rolling circle replication, a mechanism used by many plasmids and phages. Rolling circle replication generates single-stranded DNA (ssDNA). We found that ICE*Bs1*, from *Bacillus subtilis*, contains at least two regions that enable conversion of ssDNA to double-stranded DNA. At least one of these regions functions as an *sso* (single strand origin of replication). ICE*Bs1* Sso activity was important for the ability of transferred ICE*Bs1* to be acquired by recipients and for the ability of ICE*Bs1* to replicate autonomously after excising from its host’s chromosome. We identified putative *sso*'s in several other ICEs, indicating that Sso activity is likely important for the replication, stability and spread of these elements.

## Introduction

Horizontal gene transfer, the ability of cells to acquire DNA from exogenous sources, is a driving force in bacterial evolution, facilitating the movement of genes conferring antibiotic resistance, pathogenicity, and other traits [1]. Conjugation, a form of horizontal gene transfer, is the contact-dependent transfer of DNA from a donor to a recipient, generating a transconjugant. During conjugation, the DNA to be transferred is processed and protein machinery in the donor mediates transfer to the recipient. The proteins involved in DNA processing and conjugation are encoded by a conjugative element.

Integrative and conjugative elements (ICEs, also called conjugative transposons), appear to be more prevalent than conjugative plasmids [2]. The conjugation machinery (a type IV secretion system) encoded by ICEs is homologous to that of conjugative plasmids, and much of what is known about the mechanisms of transfer have come from studies of conjugative plasmids [3,4]. The defining feature of ICEs that distinguish them from conjugative plasmids is that ICEs are typically found integrated into a host chromosome and are passively propagated during chromosomal replication and cell division.

**Fig 1:**
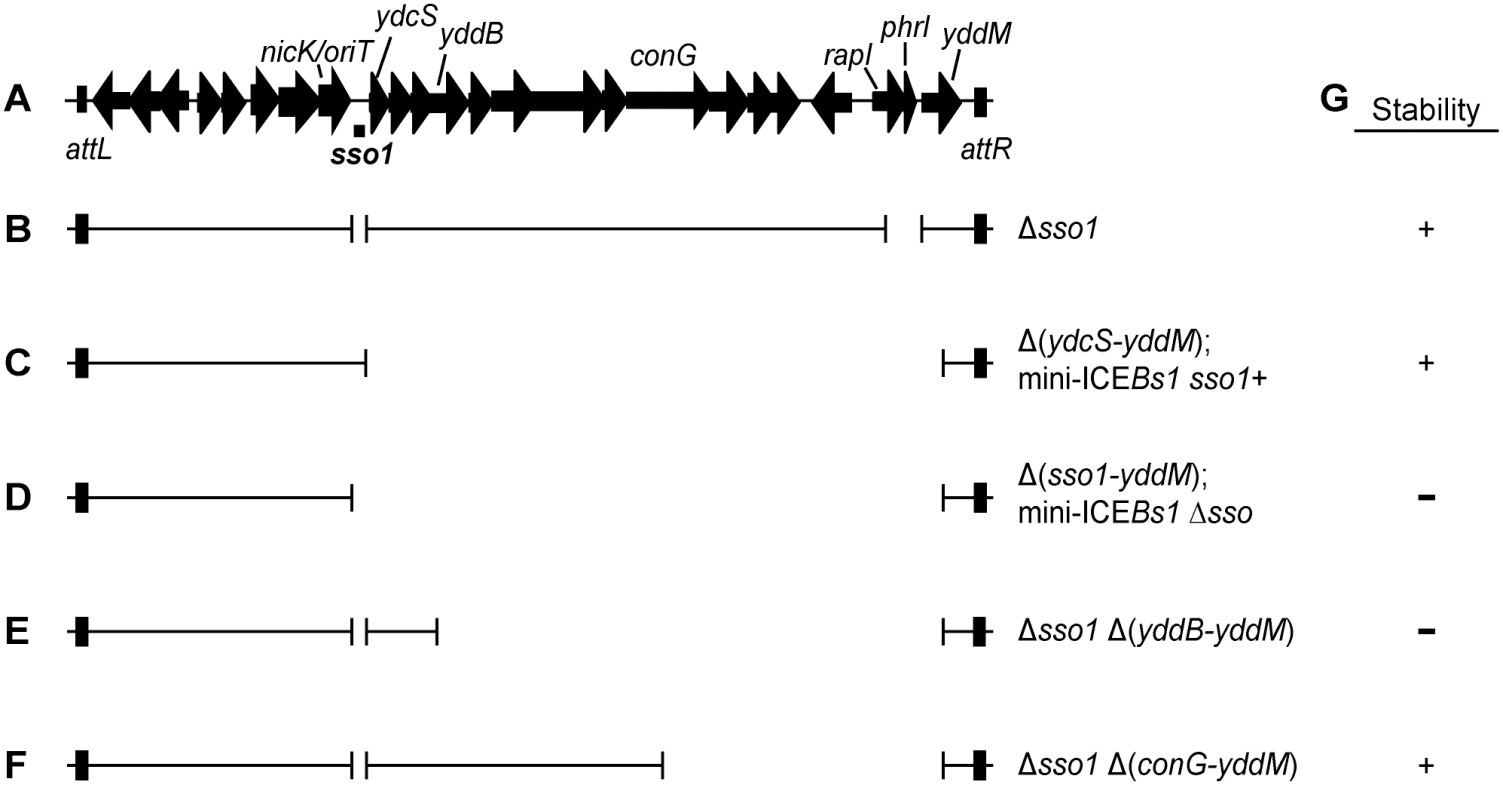
Map of ICE*Bs1* and mutants. **(A)** Map of ICE*Bs1*. A map of the genes and some of the sites in the ~20 kb ICE*Bs1* is shown, not precisely to scale. The location of *sso1*, between *nicK* and *ydcS*, is indicated underneath the map of ICE*Bs1*. This is the ~200 bp sequence that is similar to the *sso* of pTA1060 (Fig 2). The 418 bp fragment that was cloned to test for *sso* activity extends ~100 bp upstream and downstream of the region shown. Arrows indicate open reading frames and the direction of transcription. The black rectangles at the ends of ICE*Bs1* represent the flanking 60 bp repeats *attL* and *attR* that contain the site-specific recombination sites required for excision of the element from the chromosome. **(B-F)** Schematics of ICE*Bs1* mutants used to test *sso* function. Thin lines indicate the regions of ICE*Bs1* present and gaps correspond to deleted regions. Except for the markerless Δ*sso1* allele, all deletions also contain an insertion of a *kan* cassette (not included in the figure). Strains containing these ICE*Bs1* mutants are listed in Table 1. **(B)** The Δ*sso1* markerless deletion is denoted by the gap surrounding *sso1*. The larger gap near the right end indicates deletion of *rapI*-*phrI* and insertion of *kan* (not included in the figure). **(C-D)** Schematics of mini-ICE*Bs1* mutants that are missing all genes and most sequences downstream of *sso1*. **(C)** contains *sso1*; **(D)** missing *sso1*. **(E-F)** Schematics of Δ*sso1* ICE*Bs1* mutants that are also missing sequences between *yddB* and *yddM* **(E)** or *conG* and *yddM* **(F)**. Deletion endpoints are described in Materials and Methods. **(G)** Summary of the results from experiments measuring stability of the ICE*Bs1* mutants after induction in dividing host cells and in transconjugants. + indicates stable; − indicates decreased stability.

ICE*Bs1* from *Bacillus subtilis* (Fig 1) is an integrative and conjugative element that is easily manipulated and can be activated in the vast majority of cells in a population [5–10]. Like most other ICEs, ICE*Bs1* resides integrated in the host chromosome and most of its genes are repressed [5,11,12]. ICE*Bs1* gene expression is induced in response to DNA damage during the RecA-dependent SOS response and following production of the cell- sensory protein RapI [5,6,13]. DNA damage and RapI independently cause proteolytic cleavage of the ICE*Bs1* repressor ImmR [13], leading to de-repression of ICE*Bs1* gene expression and production of proteins needed for excision and transfer. Excision from the chromosome results in the formation of a circular plasmid form of ICE*Bs1*. If appropriate recipients are present, the ICE*Bs1*-encoded conjugation machinery can mediate transfer, presumably of linear single-stranded DNA (ssDNA) from host (donor) to recipient, generating a transconjugant. ICE*Bs1* then integrates into the chromosome of the transconjugant. ICE*Bs1* can be induced in >90% of cells in a population simply by overexpression of the regulator *rapI* [5,6].

The proteins that transfer ICEs are homologous to those that transfer conjugative plasmids, including the F plasmid from *Escherichia coli* [14] and pCF10 from *Enterococcus faecalis* [4]. Prior to transfer, a relaxase nicks one DNA strand at the origin of transfer (*oriT*) and becomes covalently attached to the 5' end. Based on analogy to the F and Ti plasmids, the DNA is unwound after nicking and the nicked ssDNA with the attached relaxase is transferred to the recipient [15–18]. Once in a recipient (now a transconjugant), the relaxase attached to the transferred linear ssDNA is believed to catalyze the circularization (ligation) of ssDNA with the concomitant release of the relaxase [19–21]. Conversion of this circular ssDNA to dsDNA in the transconjugant should be needed for efficient integration into the chromosome of the new host because the substrate for the ICE recombinase (integrase) is typically dsDNA [22]. Also, following the same rationale, it is likely that integration of the ICE back into the host chromosome from which it originally excised requires that the ICE be double-stranded.

The nicking and unwinding of conjugative DNA for transfer is similar to the early events in rolling circle replication (RCR) used by many plasmids and phages [reviewed in 23]. RCR plasmids encode a relaxase that binds a plasmid region called the double strand origin (*dso*) and nicks a single DNA strand (the leading strand). Following association of a helicase and the replication machinery, leading strand DNA replication proceeds from the free 3’ end using the circular (un-nicked) strand as template. Once the complement of the circular strand is synthesized, the relaxase catalyzes the release of two circular DNA species: one dsDNA circle, and one ssDNA circle.

The circular ssDNA is converted to dsDNA, typically using an RNA primer to a region of the circular ssDNA. Priming of the ssDNA circle allows DNA polymerase to synthesize the complementary strand, followed by joining of the free DNA ends by host DNA ligase [23]. Three general mechanisms for initiation of RNA primer synthesis for RCR and conjugative plasmid complementary strand synthesis have been described: 1) recruitment of host RNA polymerase; 2) recruitment of host primase; and 3) use of a plasmid-encoded primase [3,24,25]. Recruitment of the host RNA polymerase or primase typically requires a region of the plasmid that is revealed and active only when single-stranded (that is, after nicking and unwinding by helicase). This region is referred to as a single strand origin of replication (*sso*) and has been defined for many plasmids and phages that replicate by the rolling circle mechanism [24]. Sso activity also contributes to plasmid stability [26–29]. Here, we use "*sso*" to indicate a DNA sequence that is orientation-specific and promotes second strand synthesis of elements that use rolling circle replication.

Virtually nothing is known about how the transferred ssDNA of ICE*Bs1*, or any other ICE, is converted to dsDNA. During conjugation, the form of ICE*Bs1* DNA that is transferred to recipients is likely ssDNA with an attached relaxase [7,8], analogous to conjugative transfer of other elements [15–17]. Furthermore, when activated in host cells, ICE*Bs1* replicates autonomously by the rolling circle mechanism and this replication is required for stability of the excised element in a population of growing cells [30]. It is not known how dsDNA is synthesized from the ssDNA that is generated during rolling circle replication of ICE*Bs1* in host cells.

We postulated that ICE*Bs1* has an efficient mechanism for converting ssDNA to dsDNA, both during rolling circle replication of ICE*Bs1* in host cells and following conjugative transfer of ssDNA to recipients (that is, in transconjugants). Since the ICE*Bs1* relaxase does not appear to have a primase domain, we postulated that ICE*Bs1* might have a functional *sso*. We identified a single strand origin of replication in ICE*Bs1*, which we named *sso1*. *sso1* is similar to the *sso*'s of several characterized plasmids. We found that *sso1* was sufficient to correct replication defects in a plasmid that uses rolling circle replication and that otherwise did not have an *sso*, indicating that *sso1* was functional. Analyses of an *sso1* mutant of ICE*Bs1* indicated that there is at least one other region in ICE*Bs1* that is able to promote second strand synthesis. Our results indicate that Sso function in ICE*Bs1* was important for the stable acquisition of ICE*Bs1* by transconjugants and for maintenance of ICE*Bs1* following excision in host cells. These findings highlight the importance of autonomous replication in the ICE lifecycle and likely extend to many, and perhaps most, functional ICEs.

## Results

### Identification of a putative single strand origin of replication in ICE*Bs1*

**Fig 2:**
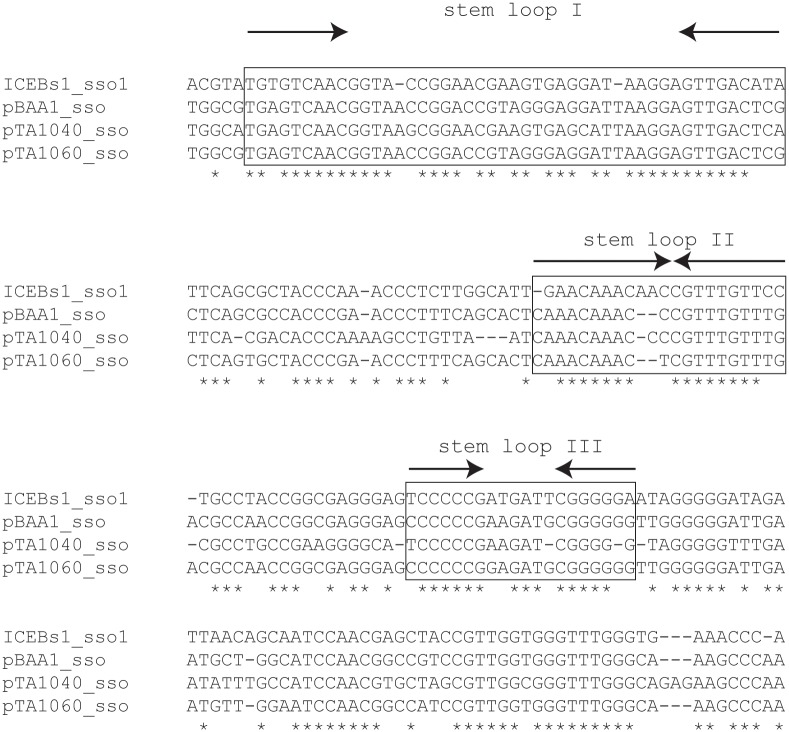
A region of ICE*Bs1* (*sso1*) is similar to *sso*'s from RCR plasmids. ICE*Bs1 sso1* is similar to the *sso*'s of *B*. *subtilis* RCR plasmids pBAA1, pTA1040 (NCBI accession NC_001764.1, position 5109–5307) and pTA1060 (accession NC_001766, position 6349–6549) [32,33]. *sso* sequences were compared with the multiple sequence alignment algorithm T-COFFEE [34]. Asterisks indicate nucleotides that are identical in all four *sso*'s. Horizontal dashes correspond to gaps. The boxed regions are predicted to form stem loop structures when single-stranded and are important for pBAA1 *sso* activity [35]. Single-stranded ICE*Bs1 sso1* is also predicted to form the three stem loops important for pBAA1 *sso* activity as determined by the ssDNA folding prediction program Mfold [36]. The arrows indicate predicted stems (inverted repeats) of stem-loop structures.

We searched [31] ICE*Bs1* for sequences that are similar to known *sso*'s from plasmids that replicate in *B*. *subtilis* by rolling circle replication. We found that an intergenic region in ICE*Bs1* immediately downstream of *nicK* (the gene for relaxase) is 76% identical to the *sso* of *B*. *subtilis* RCR plasmid pTA1060 [32,33] (Figs 1A and 2). This sequence in ICE*Bs1* is also similar to the *sso*'s of related RCR plasmids pBAA1, pTA1015, and pTA1040 [33] (Fig 2). Experiments described below demonstrate that this region of ICE*Bs1* functions as an *sso*. Therefore, we named it *sso1*.

ICE*Bs1 sso1* contained conserved features known to be important for pBAA1 *sso* activity. Functional studies of the pBAA1 *sso* defined three stem-loop structures that were important for activity [35]. Single-stranded ICE*Bs1 sso1* was predicted to form three stem-loop structures that were similar to those of pBAA1 on the levels of sequence and secondary structure (Fig 2). Based on these analyses, we predicted that *sso1* was functional.

Single strand origins are known to increase the stability of RCR plasmids. They also cause a reduction in the amount of ssDNA that accumulates from RCR plasmids. We tested the ability of *sso1* from ICE*Bs1* to function as an *sso* using four different assays, one assay for stability of an RCR plasmid and three types of assays for ssDNA.

### *sso1* from ICE*Bs1* increases the stability of the RCR plasmid pHP13

pHP13 is an *sso*-deficient RCR plasmid that replicates in *B*. *subtilis* [37]. pHP13 and other *sso*-deficient RCR plasmids are relatively unstable and lost from the population without selection for the antibiotic to which the plasmid confers resistance. However, the plasmids are stabilized if they contain a functional *sso* [e.g., in 26,38].

We found that *sso1* increased the stability of pHP13. We cloned a 418 bp region containing the putative ICE*Bs1 sso1* into pHP13, generating pHP13sso1 (pCJ44), and compared stability of plasmids with and without the putative ICE*Bs1 sso1*. A single colony of *B*. *subtilis* cells containing either pHP13 or pHP13sso1 (strains CMJ77 and CMJ78, respectively; Table 1) was inoculated into rich (LB) liquid medium containing 2.5 μg/ml chloramphenicol to select for each plasmid. Exponentially growing cultures were diluted ~1/50 in antibiotic-free LB medium. Cultures were diluted as needed in non-selective medium to maintain exponential growth. After approximately 20 generations of growth, cells were plated on antibiotic-free LB agar. Individual colonies were picked and tested for growth on selective (antibiotic-containing) and non-selective agar plates to calculate the percentage of antibiotic-resistant (plasmid-containing) clones. Approximately 11% (43/400) of the colonies tested had lost pHP13. In contrast, only 0.5% (2/400) of the colonies tested had lost pHP13sso1.

**Table 1:** *Bacillus subtilis* strains used.

Strain	Relevant genotype^1^ (reference)
IRN342	Δ(*rapI-phrI*)*342*::*kan* [5]
CAL85	ICE*Bs1* ^0^ *str-84* [5]
CAL874	Δ(*rapI-phrI*)*342*::*kan*, *amyE*::{(Pxyl-*rapI*) *spc*}[30]
CMJ77	ICE*Bs1* ^0^, pHP13 (*cat mls*)
CMJ78	ICE*Bs1* ^0^, pCJ44 (pHP13sso1 *cat mls*)
CMJ102	ICE*Bs1* ^0^, pCJ45 (pHP13sso1R *cat mls*)
CMJ118	ICE*Bs1* ^0^, *lacA*::{(PrpsF-*rpsF-ssb-mgfpmut2*) *tet*}
CMJ129	ICEBs1^0^, pHP13, *lacA*::{(PrpsF-*rpsF-ssb-mgfpmut2*) *tet*}
CMJ130	ICE*Bs1* ^0^, pCJ44 (pHP13sso1), *lacA*::{(PrpsF-*rpsF-ssb-mgfpmut2*) *tet*}
CMJ131	ICE*Bs1* ^0^, pCJ45 (pHP13sso1R), *lacA*::{(PrpsF-*rpsF-ssb-mgfpmut2*) *tet*}
LDW21	Δ(*rapI-phrI*)*342*::*kan*, *amyE*::{(Pxyl-*rapI*) *spc*}
LDW22	Δ*sso1-13*, Δ(*rapI-phrI*)*342*::*kan*, *amyE*::{(Pxyl-*rapI*) *spc*}
LDW50	Δ*sso1-13*, Δ(*conG-yddM*)*39*::*kan*, *amyE*::{(Pxyl-*rapI*) *cat*}, *thrC325*::{(ICE*Bs1-311* Δ*attR*::*tet*) *mls*}
LDW52	Δ*sso1-13*, Δ(*yddB-yddM*)*41*::*kan*, *amyE*::{(Pxyl-*rapI*) *cat*}, *thrC325*::{(ICE*Bs1-311* Δ*attR*::*tet*) *mls*}
LDW87	Δ*sso1-13*, Δ(*conG-yddM*)*39*::*kan*, *amyE*::{(Pxyl-*rapI*) *spc*}
LDW89	Δ*sso1-13*, Δ(*yddB-yddM*)*41*::*kan*, *amyE*::{(Pxyl-*rapI*) *spc*}
LDW129	Δ(*ydcS-yddM*)*93*::*kan*, *amyE*::{(Pxyl-*rapI*) *spc*}, *thrC325*::{(ICE*Bs1-311* Δ*attR*::*tet*) *mls*}
LDW131	Δ(*ydcS-yddM*)*93*::*kan*, *amyE*::{(Pxyl-*rapI*) *spc*}
LDW179	Δ(*sso1-yddM*)*177*::*kan*, *amyE*::{(Pxyl-*rapI*) *spc*}, *thrC325*::{(ICE*Bs1-311* Δ*attR*::*tet*) *mls*}
LDW180	Δ(*sso1-yddM*)*177*::*kan*, *amyE*::{(Pxyl-*rapI*) *spc*}

^1^All strains are derived from JH642 [67,68] and contain *trpC* and *pheA* mutations. These alleles are not indicated in the table.

We found that this activity of *sso1* was orientation-specific. pHP13 containing *sso1* in the reverse orientation, pHP13sso1R, (pCJ45) (strain CMJ102) was not stabilized. After approximately 20 generations of non-selective growth, 13% (13/100) of colonies tested had lost pHP13sso1R. These measurements of stability of pHP13 and derivatives are consistent with previous reports analyzing the stability of pHP13 and its *sso+* parent plasmid pTA1060 [39]. Our results are most consistent with the notion that the fragment from ICE*Bs1* cloned into pHP13 functions as an *sso*. However, our results might also indicate that the cloned sequence could function as a partitioning site, or increase plasmid copy number, or somehow stabilize the plasmid by a mechanism separate from a possible function as an *sso*.

### Visualization of *sso1* activity in live, individual cells

**Fig 3:**
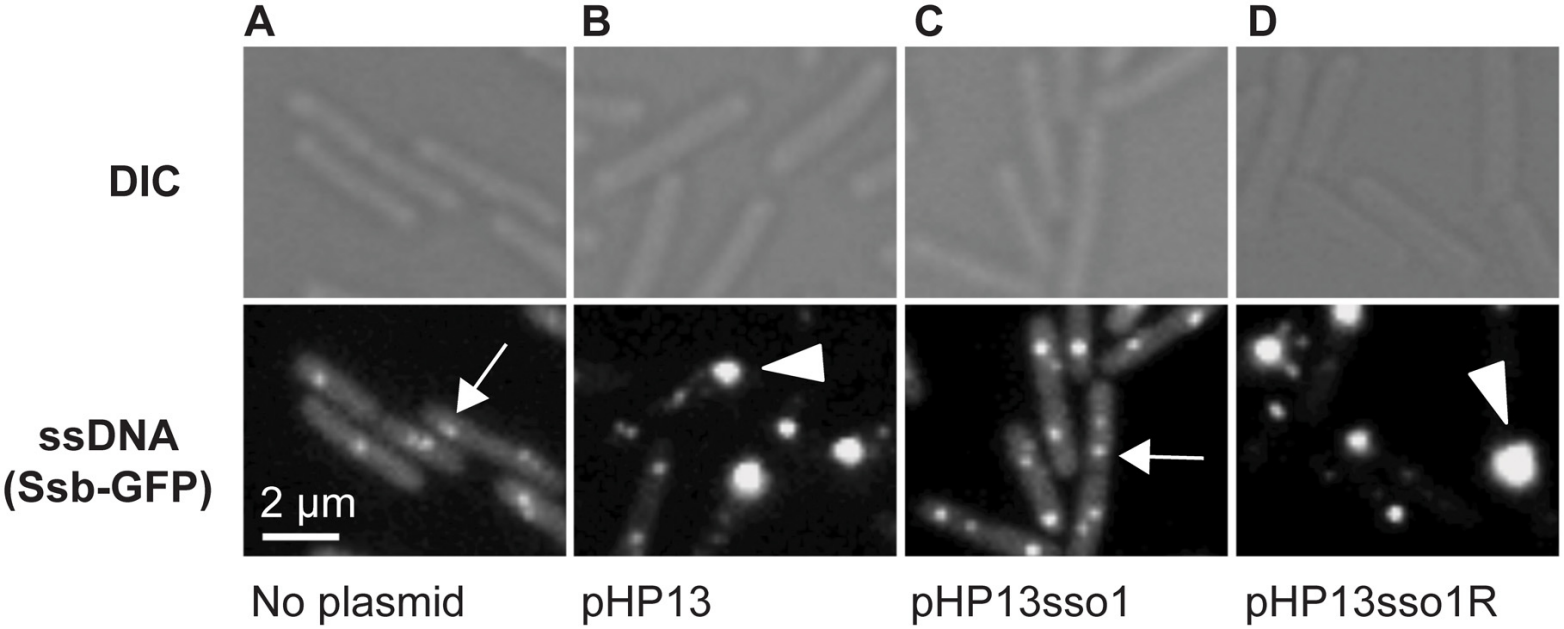
Visualization of Sso function in living cells. All cells expressed Ssb-GFP, which binds to ssDNA. Top and bottom panels are images from differential interference contrast (DIC) and fluorescence microscopy, respectively. Representative images are shown. **(A)** no plasmid, strain CMJ118. The small foci of Ssb-GFP are likely located at the replication forks [40]. One focus of Ssb-GFP is indicated with an arrow. **(B)** pHP13 (no *sso*), strain CMJ129. Large foci of Ssb-GFP foci were observed in cells that contain pHP13, a plasmid that replicates by a rolling circle mechanism but does not contain an *sso*. An arrowhead marks one large focus, indicating that Ssb-GFP likely bound to pHP13 ssDNA. **(C)** pHP13sso1, strain CMJ130. Cells containing pHP13sso1 did not have as many large foci of Ssb-GFP as those in panel B. An arrow indicates a small focus similar to those observed in cells with no plasmid (panel A). **(D)** pHP13sso1R, strain CMJ131. Cells containing pHP13 with ICE*Bs1 sso1* cloned in the reverse orientation had large foci of Ssb-GFP, indicating the accumulation of ssDNA. An arrowhead highlights a large focus, similar to the large foci observed in cells with pHP13 (no *sso*) (panel B).

To further test the function of *sso1* from ICE*Bs1*, we visualized ssDNA in living cells using a fusion of the *B*. *subtilis* single strand DNA binding protein (Ssb) to green fluorescent protein (Ssb-GFP). Cells contained either no plasmid, pHP13, pHP13sso1, or pHP13sso1R. We measured the intensity and area of the Ssb-GFP foci and calculated the percentage of cells containing at least one large intense focus (Materials and Methods). Under the growth conditions used, virtually all plasmid-free cells contained at least one focus of Ssb-GFP (Fig 3A), most likely associated with replication forks, as described previously [40,41]. Of these plasmid-free cells with foci of Ssb-GFP, approximately 5% (344 total cells observed) contained a large bright focus (evaluated using ImageJ with defined intensities described in Materials and Methods).

In contrast to the plasmid-free cells, approximately 38% of cells (of 830 total cells observed) containing pHP13 (no *sso*) had a large bright focus of Ssb-GFP (Fig 3B) in addition to the smaller foci found in plasmid-free cells. Like the plasmid-free cells, only ~4% of cells (521 total cells counted) containing pHP13sso1 had large foci of Ssb-GFP (Fig 3C). This is consistent with the expectation that there should be less ssDNA in cells with the plasmid with an *sso*. In contrast, approximately 45% of cells (of 939 total cells counted) containing pHP13sso1R had a large focus of Ssb-GFP (Fig 3D). Based on these results we suggest that the large foci were due to the accumulation of pHP13 ssDNA bound by Ssb-GFP, that ICE*Bs1 sso1* reduces accumulation of single-stranded plasmid DNA, and that the function of *sso1* is orientation specific.

### *sso1* decreases binding of Ssb-GFP to plasmid DNA

**Fig 4:**
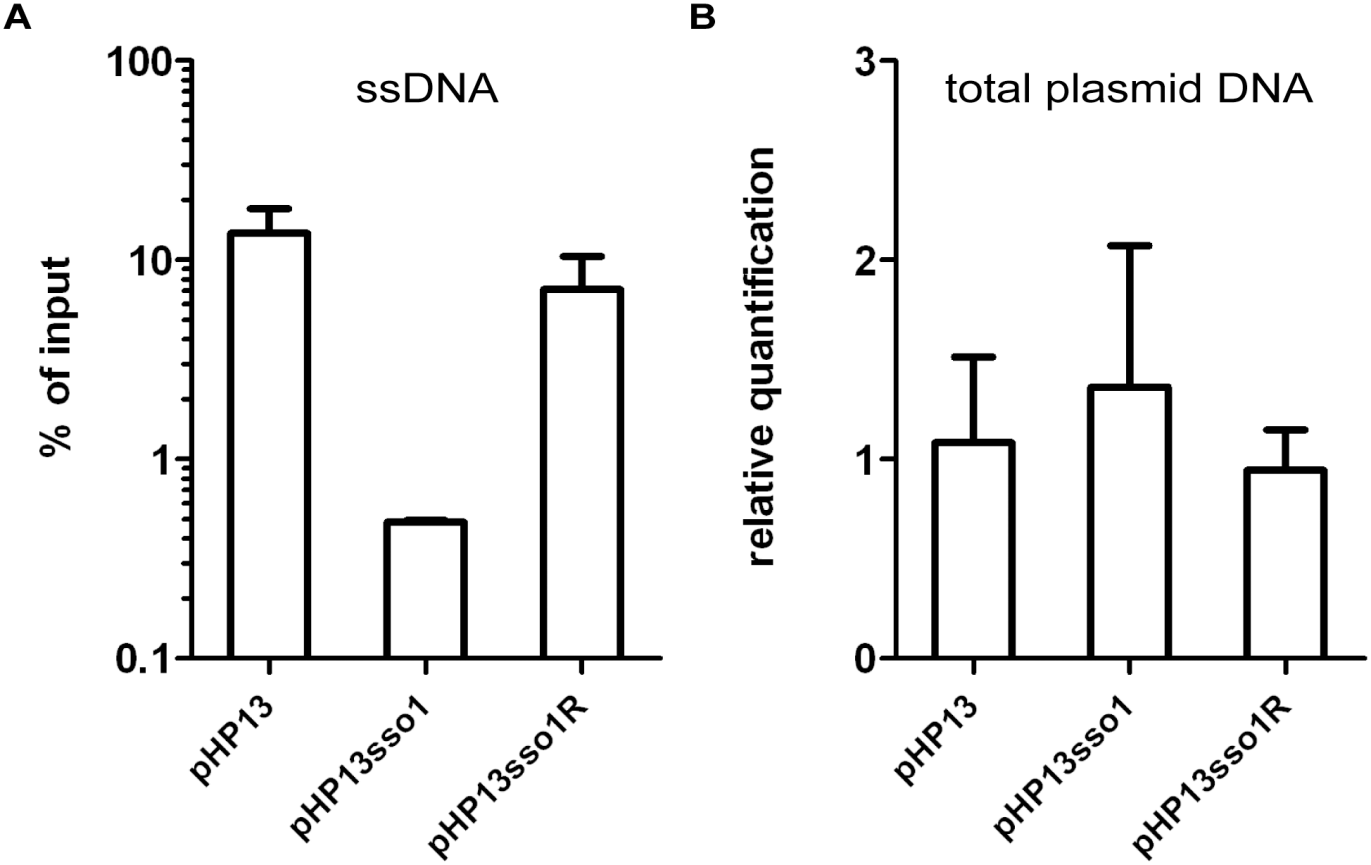
The amount of plasmid DNA associated with Ssb-GFP was decreased in the plasmid with *sso1*. We measured the relative amount of plasmid DNA associated with Ssb-GFP **(A)** and the relative amount of total plasmid **(B)**, for plasmids with and without *sso1*. Strains containing the indicated plasmid were: CMJ129 (pHP13); CMJ130 (pHP13sso1); and CMJ131 (pHP13sso1R). **(A)** Association of plasmid DNA with Ssb-GFP was measured by chromatin immunoprecipitation (ChIP) with polyclonal anti-GFP followed by qPCR with primers to detect *cat* in pHP13. The plasmid qPCR signal from immunoprecipitated DNA was normalized to the qPCR signal in pre-immunoprecipitation lysates (% of input) [42]. Data are from one representative experiment of 6 independent experiments. Error bars indicate the standard deviation of technical triplicates. **(B)** Relative quantification of plasmid DNA was determined by qPCR. Plasmid abundance (pHP13 *cat*) was normalized to a control chromosomal locus (*ydbT*), and the abundance of pHP13sso1 and pHP13sso1R was normalized relative to pHP13. Data are averages from 5 independent experiments ± standard deviation.

We verified that Ssb-GFP was bound to plasmid DNA using chromatin immunoprecipitation followed by quantitative PCR (ChIP-qPCR). We crosslinked protein and DNA using formaldehyde, immunoprecipitated Ssb-GFP with anti-GFP antibodies, and measured plasmid DNA in the immunoprecipitates with PCR primers specific to part of pHP13 (Materials and Methods). We found that the relative amount of plasmid DNA associated with Ssb-GFP was 25-30-fold greater in cells containing pHP13 (no *sso*) than that in cells containing pHP13sso1 (Fig 4A). This activity of *sso1* was also orientation specific as the amount of pHP13sso1R DNA associated with Ssb-GFP was similar to that of pHP13 (without an *sso*). The inserts did not significantly alter the relative quantity of plasmid DNA in medium containing antibiotic (Fig 4B), consistent with previous analyses of pHP13-derived plasmids [39].

### *sso1* reduces the proportion of single-stranded plasmid DNA

To further test the function of *sso1*, we used Southern blots to compare the amounts of single and double-stranded plasmid DNA in cells containing pHP13, pHP13sso1, and pHP13sso1R. An RCR plasmid without an *sso* generates a greater fraction of ssDNA than the same plasmid with a functional *sso* [29] The approach to distinguish dsDNA and ssDNA is to compare two Southern blots, one in which the DNA is denatured, and the second in which the DNA is not denatured, prior to transfer from gel to membrane. Both dsDNA and ssDNA are detected in the blot that was denatured whereas only ssDNA is detected in the blot that was not denatured. The probe used to detect the plasmids was a ^32^P-labeled ~1 kb DNA fragment complementary to *cat* in pHP13. The probe was labeled on the strand complementary to the template strand for second strand (*sso1*-driven) synthesis.

**Fig 5:**
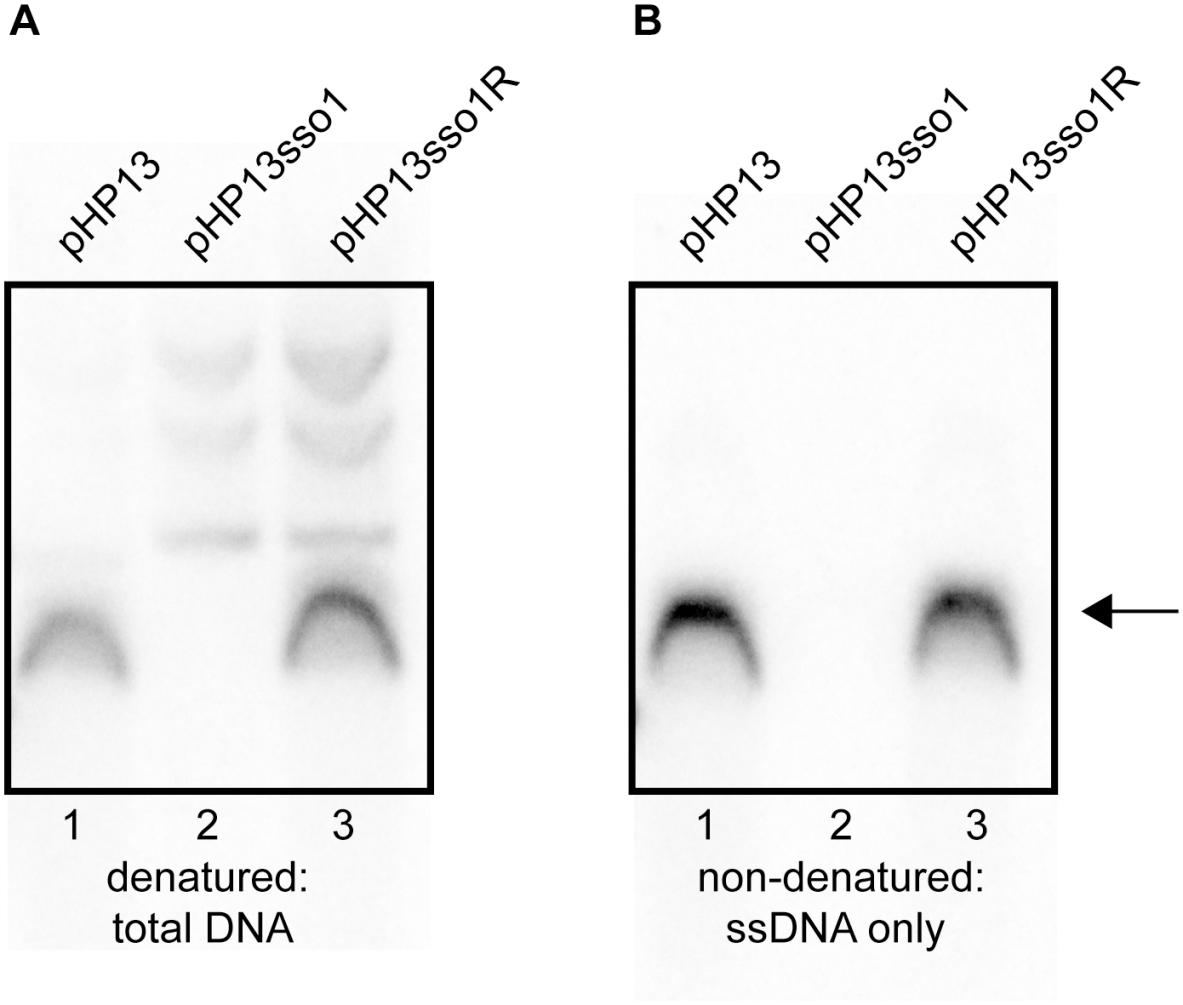
Southern blot analysis demonstrates that *sso1* decreases the amount of plasmid ssDNA. The relative amounts of plasmid DNA were determined for the indicated plasmids. Strains containing the plasmids were: CMJ77 (pHP13); CMJ78 (pHP13sso1); and CMJ102 (pHP13sso1R). The probe was ^32^P-labeled ssDNA from *cat* in pHP13 (Materials and Methods). The arrow to the right indicates single-stranded plasmid DNA. All three plasmids produced slower-migrating bands that were only detected when DNA was denatured, although these bands are faint for pHP13 in the exposure and experiment shown. Similar results were obtained in at least three independent experiments. **(A)** DNA on the filters was denatured and then probed for plasmid sequences. DNA that was either double-stranded or single-stranded before blotting is detected. **(B)** DNA on the filter was not denatured. In this blot, only DNA that was single-stranded before transfer to the filter is detected. ssDNA from pHP13sso1 was not readily detected in the Southern blots, but was detected in the ChIP-qPCR experiments with Ssb-GFP (Fig 4). This is likely due to amplification from PCR in the ChIP experiments.

We detected plasmid DNA in Southern blots with DNA that had been denatured prior to transfer to membranes (Fig 5A). There was one major DNA species from cells containing pHP13 (Fig 5A, lane 1) or pHP13sso1R (Fig 5A, lane 3). This species was not detectable in cells containing pHP13sso1 (Fig 5A, lane 2). However, slower-migrating DNA bands were detected from pHP13sso1-containing cells (Fig 5A, lane 2).

To measure single-stranded plasmid DNA, we probed DNA that had not been denatured before transfer. As expected, cells containing pHP13 had readily detectable levels of single-stranded plasmid DNA (Fig 5B, lane 1). This band corresponded to the major band observed in the denaturing blot (Fig 5A, lane 1). In contrast, cells containing pHP13sso1 had barely detectable levels of single-stranded plasmid DNA (Fig 5B, lane 2), consistent with a drop in the proportion of ssDNA of the plasmid with the *sso*. pHP13sso1 plasmid DNA was detectable under denaturing conditions (Fig 5A, lane 2), demonstrating that the low signal of pHP13sso1 ssDNA was not due to lack of plasmid DNA in the sample. The effect of *sso1* on ssDNA content was orientation specific as cells with pHP13sso1R had single-stranded plasmid (Fig 5B, lane 3), comparable to that detected in the denaturing blot (Fig 5A, lane 3).

These data indicate that ICE*Bs1 sso1*, when present on pHP13, reduced accumulation of the ssDNA replication intermediate of pHP13. The findings are consistent with the analyses of Ssb-GFP foci and association with plasmid DNA. Based on this combination of data, we conclude that *sso1* from ICE*Bs1* is a functional single strand origin of replication that enables second strand DNA synthesis to an RCR plasmid in an orientation-specific manner.

### Deletion of *sso1* in ICE*Bs1* and an additional region that is redundant with *sso1*

To test the function of *sso1* in the context of ICE*Bs1*, we constructed a deletion of *sso1* (Fig 1B, Δ*sso1*) and measured conjugation efficiency. Disappointingly, the conjugation frequency of ICE*Bs1* Δ*sso1* was indistinguishable from that of ICE*Bs1 sso1*+ (~1% transconjugants per donor for both ICE*Bs1* Δ*sso1* and ICE*Bs1 sso1*+). This result could indicate that *sso1* does not function during ICE*Bs1* conjugation, or that there is at least one other way to convert ssDNA to dsDNA in transconjugants.

If there are other regions of ICE*Bs1* that provide the ability to synthesize the second strand of DNA, then removal of such regions should uncover a phenotype for *sso1* in ICE*Bs1*. We found that removal of ICE*Bs1* DNA from *ydcS* to *yddM* (Fig 1) revealed the role of *sso1* in the ICE*Bs1* life cycle. We made two versions of this deletion derivative of ICE*Bs1*, one with and one without *sso1*, referred to as mini-ICE*Bs1 sso1*+ (Fig 1C) and mini-ICE*Bs1 Δsso1* (Fig 1D). These mini-ICEB*s1*'s contain the known regulatory elements in the left end (Fig 1A), the origin of transfer (*oriT*) and ICE*Bs1* genes needed for nicking and replication, and genes and sites needed for excision and integration. The mini-ICE*Bs1* is functional for excision and can transfer to recipient cells using transfer functions provided in trans from a derivative of ICE*Bs1* that cannot excise or transfer [6]. Experiments described below (summarized in Fig 1G) indicate that single strand origin function is important for both stable acquisition by transconjugants and for maintenance in host cells following excision from the chromosome.

### *sso1* contributes to stable acquisition of ICE*Bs1* by transconjugants

We tested the ability of mini-ICE*Bs1 sso1+* and mini-ICE*Bs1* Δ*sso1* to be stably acquired by recipients in conjugation. We mated mini-ICE*Bs1 sso1*+ (encoding kanamycin resistance) into a wild type recipient (streptomycin resistant), selecting for resistance to kanamycin and streptomycin. Donor cells also contained a derivative of ICE*Bs1* integrated at *thrC* that is able to provide conjugation functions, but that is unable to excise and transfer [6]. In these experiments, the mini-ICE*Bs1* is mobilized by the transfer machinery encoded by the non-excisable element at *thrC*. Recombination between the element at *thrC* and the mini-ICE*Bs1* would result in loss of *kan* from the mini-ICE*Bs1* and would not yield kanamycin-resistant transconjugants.

**Fig 6:**
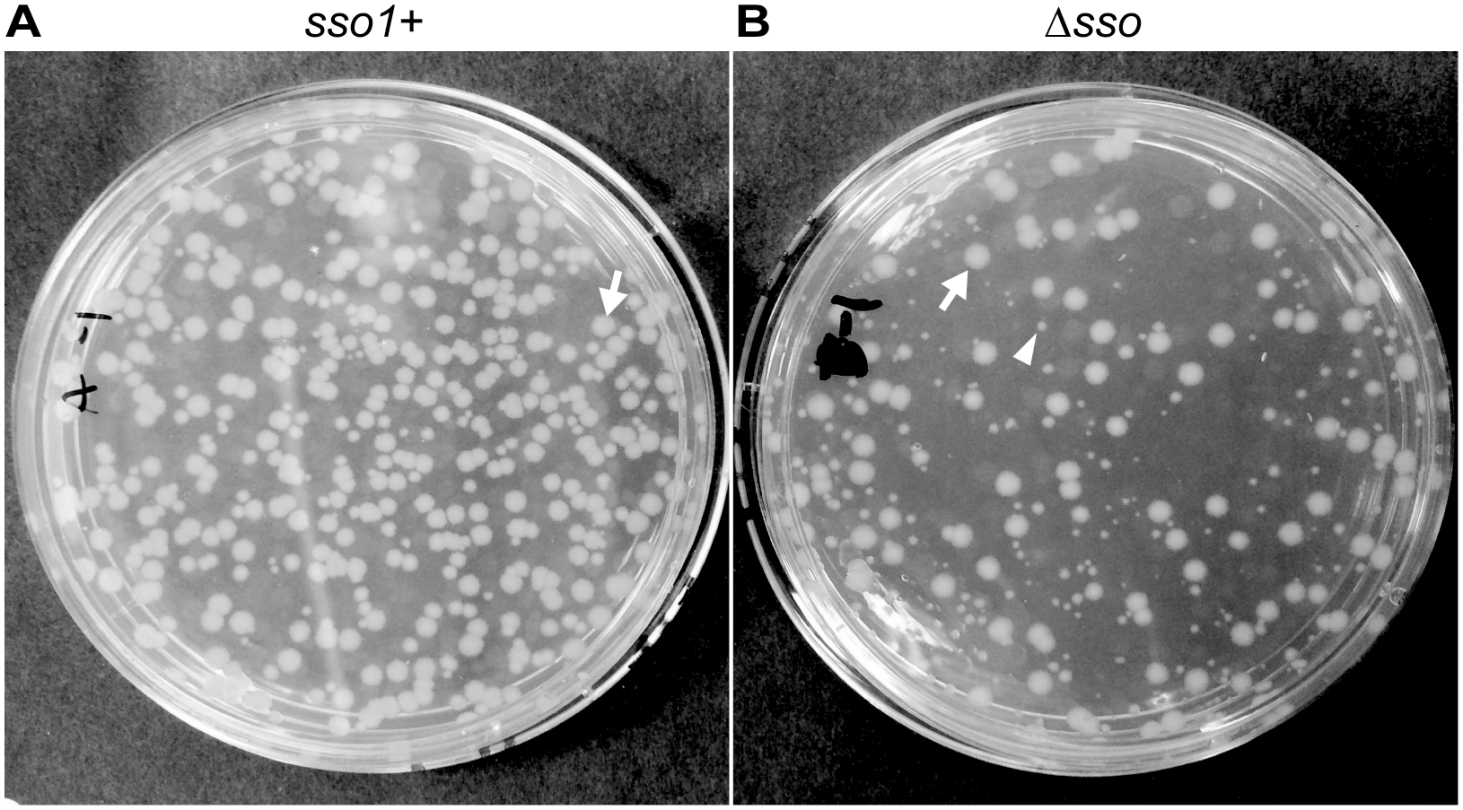
*sso1* contributes to stable acquisition of ICE*Bs1* by recipients. Mini-ICE*Bs1* with (**A**; strain LDW129) or without (**B**; strain LDW179) *sso1* was crossed into recipients (strain CAL85) by conjugation. Transconjugants were selected on solid medium containing streptomycin and kanamycin. The conjugation frequency (~0.2%) is about one tenth that for wild type ICE*Bs1*. The non-excisable element at *thrC* is a substrate for nicking and RCR [30,43] and produces relaxosome complexes that we suspect compete with and reduce the efficiency of transfer of the mini-ICE*Bs1*. In addition, nicking and replication from non-excisable elements kills donor cells [43]. **(A)** Transconjugants that acquired mini-ICE*Bs1 sso1*+ grew as relatively uniform, normal looking colonies. Transconjugants (including those that initially grew smaller than normal) were resistant to kanamycin after propagation under non-selective conditions, indicating that ICE*Bs1* was stably acquired (as described in the text). **(B)** Transconjugants that acquired mini-ICE*Bs1* Δ*sso1* produced at least two types of colonies, large and small. An arrow marks one large (normal) colony, and an arrowhead indicates one small colony. Most small colonies were unstable. That is, after propagation without selection, cells derived from small colonies were no longer resistant to kanamycin, indicating that ICE*Bs1* was not stably acquired (as described in the text).

The conjugation (mobilization) efficiency of mini-ICE*Bs1 sso1*+ was ~0.2% transconjugants per donor. Transconjugants on selective medium produced normal looking colonies (Fig 6A) that stably maintained kanamycin resistance even after propagation under non-selective conditions. We picked 50 transconjugants, streaked each on nonselective plates (LB agar, no antibiotic) to single colonies, and then picked a single colony from each isolate and restreaked, testing for resistance to kanamycin (LB agar with kanamycin). Each isolate tested (50/50) was still resistant to kanamycin. These results indicate that mini-ICE*Bs1 sso1*+ is transferred and stably maintained in the transconjugants, analogous to the properties of wild type ICE*Bs1*.

Results with donor cells containing mini-ICE*Bs1* Δ*sso1* (strain LDW179) were different from those containing mini-ICE*Bs1 sso1*+. The apparent conjugation (mobilization) efficiency was similar to that for mini-ICE*Bs1 sso1*+, ~0.3% transconjugants per donor. However, there were at least two types of colonies, large and small, on the original plates (LB with kanamycin and streptomycin) selective for transconjugants (Fig 6B). The large colonies were similar in appearance to the transconjugants from mini-ICE*Bs1 sso1*+ (Fig 6A).

In contrast to the transconjugants with the mini-ICE*Bs1 sso1*+, many of the transconjugants receiving mini-ICE*Bs1* Δ*sso1* appeared to be unable to stably retain this element. That is, these transconjugants were no longer resistant to kanamycin after growth under non-selective conditions. We picked all the transconjugants (153) from an LB agar plate containing kanamycin and streptomycin, streaked for single colonies on non-selective plates (LB agar without antibiotics), and then tested a single colony from each of these for resistance to kanamycin. Of the 153 isolates tested, 86 (56%) were sensitive and 67 (46%) were resistant to kanamycin. Usually, but not always, the small colonies generated cells that were sensitive to kanamycin and had apparently lost mini-ICE*Bs1 Δsso1*. The larger colonies typically generated cells that were resistant to kanamycin, indicting the stable presence of mini-ICE*Bs1 Δsso1*. These results indicate that the mini-ICE*Bs1 Δsso1* was unstable in >50% of the transconjugants.

We postulate that the mini-ICE*Bs1* Δ*sso1* is unstable in the small colonies because it is unable to integrate before the initial transconjugants grow and divide. Furthermore, we postulate that there was some conversion of ssDNA to dsDNA independent of *sso1* such that there was integration in some of the transconjugants. In addition, it seems likely that the initial kanamycin resistance of the transconjugants was due to expression of the kanamycin resistance gene (*kan*), and presumably the gene must be double-stranded to be efficiently transcribed. If this is true, it implies that integration takes time and that there is considerable cell growth and division before the mini-ICE*Bs1* Δ*sso1* can integrate. It is also possible, although we believe unlikely, that the mini-ICE*Bs1* Δ*sso1* is not converted to dsDNA. In this case, there is some other mechanism for the transconjugants to be initially resistant to kanamycin and for integration of single-stranded ICE*Bs1* DNA, perhaps by a relaxase- [19] or integrase- [44,45] mediated recombination event.

Our results indicate that the conversion of ICE ssDNA to dsDNA in transconjugants is important for the stable acquisition of the element. The initial molecular events in the recipient likely include entry of the linear single-stranded ICE DNA with the relaxase covalently attached to the 5' end, followed by relaxase-mediated circularization of the ssDNA. The presence of *sso1* on this circular ssDNA likely enables efficient synthesis of a primer for second strand DNA synthesis. In the absence of *sso1*, second strand synthesis is likely less efficient, leading to loss of ICE*Bs1* from many of the cells. ICEs that are transferred by a type IV secretion system are all thought to enter the recipient as linear ssDNA with an attached relaxase [4]. If true, then an efficient mechanism for priming second strand DNA synthesis is likely to be critical for the stable propagation and spread of these elements.

### *sso1* contributes to stability and replication of ICE*Bs1* in host cells following excision

After induction of ICE*Bs1* gene expression and excision from the chromosome, it takes several generations to reestablish repression and for re-integration into the chromosome [30]. Therefore, after excision from the chromosome, autonomous replication of ICE*Bs1* is required for its stability in host cells during growth and cell division [30].

**Fig 7:**
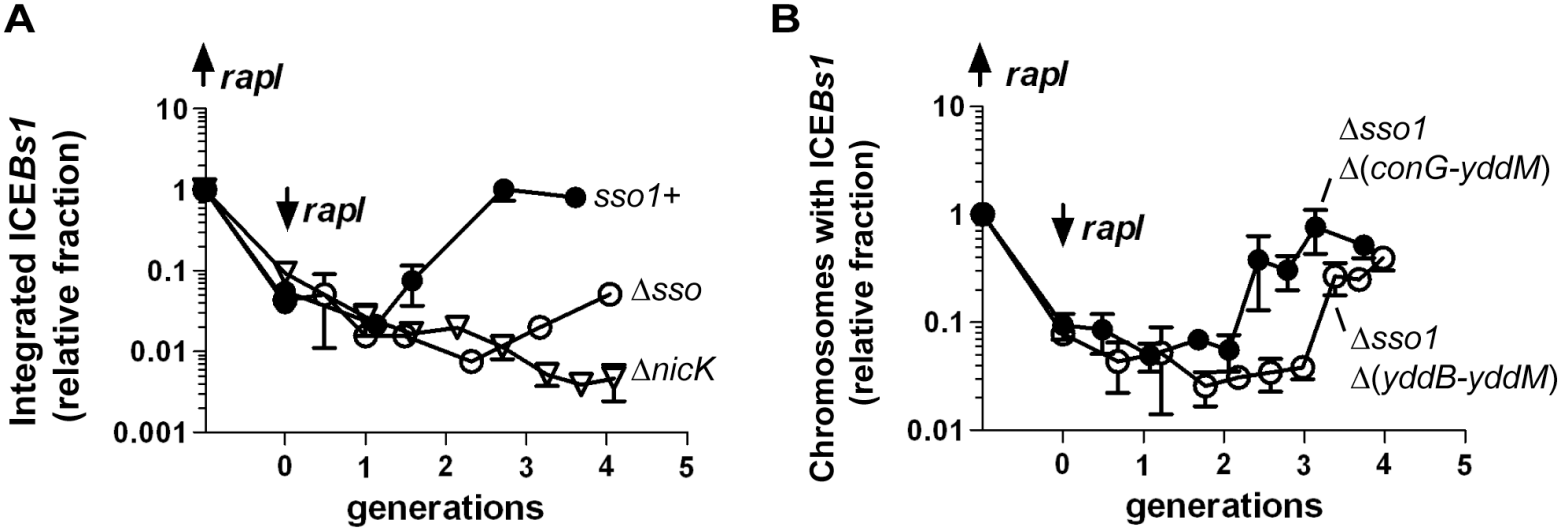
Sso activity is important for maintenance of ICE*Bs1* after excision in growing cells. The relative fraction of cells with ICE*Bs1* integrated in its attachment site in the chromosome is plotted versus the number of generations after repression of Pxyl-*rapI*. Cells were grown in defined minimal medium with arabinose as carbon source. Xylose was added during mid-exponential phase to induce expression of Pxyl-*rapI* (origin on y-axis; up *rapI*), thereby causing induction and excision of ICE*Bs1*. In all cases, ≥90% of cells had ICE*Bs1* excised from the chromosome two hours after induction of Pxyl-*rapI*. Two hours after addition of xylose, cells were pelleted and resuspended in glucose to repress expression of Pxyl-*rapI* (time = 0 generations; down *rapI*). Samples were taken for determination of the relative fraction of cells with ICE*Bs1* integrated in the chromosome generating *attL*, the junction between chromosomal sequences and ICE*Bs1*. Data presented are from one representative experiment of at least three independent experiments. Error bars indicate the standard deviation of technical triplicates. **(A)** Data for mini-ICE*Bs1* with (*sso1*+; filled circles; strain LDW131) or without (*Δsso*; open circles; strain LDW180) *sso1*. Δ*nicK* (open triangles; strain CAL1215) is an ICE*Bs1* mutant that is unable to replicate autonomously and is lost from the population of cells after excision from the chromosome and continued cell growth and division [30]. **(B)** Data are for the indicated deletion derivatives of ICE*Bs1*, both missing *sso1* and the indicated regions: Δ*sso1* Δ(*conG-yddM*) (filled circles; strain LDW87) and the derivative missing a bit more of ICE*Bs1*, Δ*sso1* Δ(*yddB-yddM*) (open circles; strain LDW89). Maps of the ICE*Bs1* mutants are in Fig 1.

We tested the contribution of *sso1* to the stability of ICE*Bs1* in host cells following excision from the chromosome. We induced gene expression and excision of mini-ICE*Bs1 sso1*+, mini-ICE*Bs1* Δ*sso1*, and replication-defective ICE*Bs1* Δ*nicK* by expressing *rapI* from a xylose-inducible promoter (Pxyl-*rapI*). By two hours after expression of *rapI*, all three derivatives of ICE*Bs1* had excised normally as indicated by a >90% decrease in *attL*, the junction between the left end of ICE*Bs1* and chromosomal sequences (Fig 7A). At that time (two hours post-induction, 0 generations in Fig 7), *rapI* expression was repressed by removing xylose and adding glucose. We then monitored the kinetics of reintegration of mini-ICE*Bs1 sso1*+, mini-ICE*Bs1* Δ*sso1*, and ICE*Bs1* Δ*nicK* into the host chromosome using qPCR to monitor the formation of *attL*. If the plasmid form of the element cannot replicate, then we expect a decrease in the proportion of cells that contain an integrated element (a decrease in formation of *attL*) compared to that in cells with an element that can replicate. After ~4 generations (6 hours of *rapI* repression), mini-ICE*Bs1 sso1*+ had reintegrated into the chromosome in ~80% of cells (assuming 100% integration before induction of ICE*Bs1* gene expression). In contrast, mini-ICE*Bs1* Δ*sso1* had reintegrated in ~5% of the cells (Fig 7A). This amount of integration was significantly less than that of mini-ICE*Bs1 sso1*+, but greater than that of ICE*Bs1* Δ*nicK* (Fig 7A), indicating that mini-ICE*Bs1* Δ*sso1* is inefficiently maintained in dividing host cells but that it is more stable than the ICE*Bs1* mutant that is completely unable to replicate.

We also determined the fraction of colony forming units (CFUs) that were kanamycin-resistant after four generations of growth. Cells were plated non-selectively (without antibiotic) and then individual colonies picked and tested for resistance to kanamycin. Since each of the ICE*Bs1* derivatives contains *kan*, this should be a good indication of stable integration of ICE*Bs1*. Consistent with qPCR results, mini-ICE*Bs1 sso1*+ was present in >95% (36/37) of cells (CFUs, plated at generation = 4) as judged by resistance to kanamycin. In contrast, <5% of cells (1/25) plated after 4 generations contained mini-ICEBs1 Δ*sso1*. That is, only one of the 25 colonies tested had robust growth on kanamycin. Of the 24 cells that did not form robust colonies, 12 did not have any detectable growth, and 12 grew poorly on kanamycin. Since ICE*Bs1* DNA must presumably be double-stranded in order to integrate into the chromosome by Int-mediated site-specific recombination, and based on the results above showing that *sso1* functions as a single strand origin of replication in a plasmid (pHP13), the simplest model is that *sso1* also functions as a single strand origin of replication in the mini-ICE*Bs1*, and in ICE*Bs1*.

### ICE*Bs1* DNA between *yddB* and *conG* is functionally redundant with *sso1*

Results presented above indicate that, in the context of mini-ICE*Bs1*, *sso1* has a function and causes a phenotype. However, in the context of an intact ICE*Bs1*, loss of *sso1* caused little if any detectable phenotype. Together, these results indicate that there is a region downstream of *sso1* in ICE*Bs1* that somehow enables synthesis of the second strand of DNA. We used derivatives of ICE*Bs1* with different amounts of DNA downstream from *sso1* to determine the location of at least one of these regions.

We found that the region between *yddB* and *conG* (Fig 1A) was at least partly functionally redundant with *sso1*. We compared the deletion derivative ICE*Bs1* Δ*sso1* Δ(*yddB-yddM*) that is missing *sso1* and sequences from *yddB* through *yddM* (Fig 1E) to ICE*Bs1* Δ*sso1* Δ(*conG-yddM*) that is missing *sso1* and sequences from *conG* through *yddM* and contains the sequences from *yddB* through *conG* (Fig 1F). As above, we measured the ability of these elements to function in conjugation and to reintegrate in host cells following induction of gene expression and excision.

#### Stability in transconjugants

We compared transconjugants that acquired ICE*Bs1* Δ*sso1* Δ(*conG-yddM*) (containing ICE*Bs1* DNA from *yddB-conG*; Fig 1F) to those that acquired ICE*Bs1* Δ*sso1* Δ(*yddB-yddM*) (containing ICE*Bs1* DNA upstream from *ydcS*-*yddB*; Fig 1E). Transconjugants that acquired ICE*Bs1* Δ*sso1* Δ(*conG-yddM*) were stable and produced relatively uniform colonies. In contrast, transconjugants that acquired ICE*Bs1* Δ*sso1* Δ(*yddB-yddM*) (Fig 1E) were unstable and colonies were heterogeneous, similar to transconjugants acquiring mini-ICE*Bs1 Δsso1*. Transconjugants acquiring each ICE*Bs1* mutant were picked, purified non-selectively, and then tested for resistance to kanamycin, indicative of the presence of the mutant ICE*Bs1*. The ICE*Bs1* Δ*sso1* derivative that contained DNA through part of *conG* {ICE*Bs1* Δ*sso1* Δ(*conG-yddM*)} was stably acquired. None of the 72 transconjugants tested had lost resistance to kanamycin after passage non-selectively. In contrast, 21 of 115 transconjugants tested that had initially acquired ICE*Bs1* Δ*sso1* Δ(*yddB-yddM*) were sensitive to kanamycin after passage non-selectively. That is, 18% had not stably maintained this mutant version of ICE*Bs1*. This mutant appeared more stable than the mini-ICE*Bs1 Δsso1*, but was less stable than the ICE*Bs1 Δsso1* that contains sequences from *yddB-conG*.

#### Stability in hosts following excision

We also tested the ability of each of these mutant versions of ICE*Bs1* to reintegrate in host cells following excision. ICE*Bs1* Δ*sso1* Δ(*conG-yddM*) (containing *yddB-conG*) was relatively stable as determined by qPCR (Fig 7B). In addition, ~94% of cells (50/53) were resistant to kanamycin four generations after repression of *rapI* (induction of *rapI* was used to induce ICE*Bs1* gene expression and excision). In contrast, integration of ICE*Bs1* Δ*sso1* Δ(*yddB-yddM*) was delayed relative to that of ICE*Bs1* Δ*sso1* Δ(*conG-yddM*) (containing *yddB-conG*) (Fig 7B). In addition, by four generations after repression of *rapI*, fewer than half of the cells (15/34) were resistant to kanamycin, indicating that only ~44% of the cells had stably integrated this mutant ICE*Bs1*. Based on these results we infer that one or more regions between *yddB* and *conG* likely enables the conversion of ICE*Bs1* from ssDNA to dsDNA, a function at least partly redundant with that of *sso1*.

We tested for Sso activity in this region by cloning DNA encompassing *yddB* to *conG*, and smaller fragments between *ydcS* and *conG*, into pHP13 (lacks an *sso*). None of the fragments tested had a functional *sso*; that is, none of the regions tested enhanced second strand synthesis or stability of pHP13. Thus, this region functions differently than *sso1*. It is possible that this region functions as an *sso* only in context of *oriT* and ICE*Bs1* and not in pHP13, perhaps by requiring another region in ICE*Bs1* for function. It is also possible that an ICE*Bs1* gene product is needed for this region to enable second strand synthesis. We have not further explored these possibilities.

### Single strand origins in other ICEs

Our results indicate that *sso1* in ICE*Bs1* is functional. Based on the life cycle of ICEs, it seems likely that many (most) other functional ICEs also have at least one region capable of functioning as an Sso. Although there is relatively little sequence similarity between *sso*'s from different plasmids and other RCR elements [24], we found that the *sso* of the RCR plasmid pC194 [46] from *Staphylococcus aureus* is identical to a region in several different ICEs from clinical isolates of *Streptococcus pneumonia* (Table 2). In addition, we found that an ICE from *Mycoplasma fermentans* has a region similar to the *sso* from the plasmid pT181 from *S*. *aureus* [47] and an ICE from *Streptococcus suis* has a region similar to the *sso* from the plasmid pUB110 from *S*. *aureus* [48]. The simplest notion is that each of these sequences functions as an *sso* for the cognate ICE.

**Table 2:** ICEs with regions identical or similar to known single strand origins from plasmids.

ICE^a^	organism	Accession#^b^	plasmid^c^	% Identity
Tn*5253*	*S*. *pneumoniae* DP1322/BM6001	EU351020.1	pC194	100%
Tn*1311*	*S*. *pneumoniae* SpnF21	FN667862.2	pC194	100%
ICE*6094*	*S*. *pneumoniae* Pn19	FR670347	pC194	100%
ICE*Spn11930*	*S*. *pneumoniae* 11930	FR671403	pC194	100%
ICE*Sp23FST81*	*S*. *pneumoniae* Sp264	FM211187	pC194	100%
ICE*F-II*	*Mycoplasma fermentans* PG18	AY168957	pT181	68%
ICE*Ssu*(BM407)2	*Streptococcus suis* BM407	FM252032	pUB110	78%

^a^ICEs with regions similar to known single strand origins in RCR plasmids were identified using searches with BLASTN [80]. Tn*5253* and Tn*1311* were identified by BLASTN against the NCBI nucleotide collection database. All other ICEs were identified using WU-BLAST 2.0 against the ICEberg v1.0 ICE nucleotide sequence database [81].

^b^Accession numbers refer to the nucleotide sequence files in GenBank.

^c^The *sso* sequences from pC194 (168 nucleotides) and pT181 (234 nucleotides) [29] and pUB110 (340 nucleotides) [82] were used to search for similar sequences in ICEs.

In contrast to the examples of sequence similarity (identity) described above, the function of an *sso* likely depends on its structure in addition to or instead of its primary sequence. For example, replacement of the *sso* of pBAA1 with a different primary sequence that retained secondary structure resulted in a functional *sso* [35]. Identification of *sso*'s in other ICEs will likely require a combination of analyses of sequence and predicted structures and direct functional tests.

## Discussion

We identified *sso1*, a functional single strand origin of replication in ICE*Bs1*. *sso1* was cloned into pHP13, a plasmid without an *sso*, and *sso1* increased pHP13 stability. Furthermore, *sso1* decreased accumulation of pHP13 ssDNA. Live cell imaging and ChIP-PCR experiments with Ssb-GFP indicated that there was less ssDNA from pHP13sso1 than from pHP13. Results from Southern blotting also revealed that the presence of *sso1* in pHP13 caused a decrease in the amount of pHP13 ssDNA. Together, these results demonstrate that *sso1* from ICE*Bs1* is a functional single strand origin of replication.

Genetic analyses of ICE*Bs1* showed that loss of *sso1* and an additional region between *yddB* and *conG* led to significant defects in ICE*Bs1* physiology consistent with impaired replication. Specifically, deletion of these regions significantly decreased maintenance of the plasmid form of ICE*Bs1*, thereby affecting 1) stability of ICE*Bs1* in host cells during growth and cell division; and 2) stable acquisition of ICE*Bs1* by transconjugants. Based on this functional redundancy, the simplest interpretation is that the region between *yydB* and *conG* somehow contributes to second strand synthesis, although it is currently not known how.

### Sso function in ICE biology

RCR plasmids and ICEs share many functional properties. Like ICE*Bs1* [30] and members of the SXT/R391 family from Gram-negative bacteria [49], other ICEs may be capable of autonomous replication via a rolling circle mechanism {[30,49] and references therein}. In addition, the ICE*Bs1 oriT* and its conjugative relaxase NicK also serve as double-stranded origin and a replicative relaxase, respectively, supporting autonomous rolling circle replication. Furthermore, some RCR plasmid replicative relaxases can serve as conjugative relaxases [50]. We have now shown an additional similarity between RCR plasmids and ICEs: the importance of Sso activity in stability of the element.

Functional ICEs appear to have a conserved lifecycle. Therefore, we suspect many other ICEs contain a single strand origin of replication to support both autonomous ICE replication in host cells and stable establishment in transconjugants. Preliminary bioinformatic analyses revealed that several ICEs contain sequences with high identity to characterized *sso*'s from RCR plasmids (Table 2). These findings support the notion that many ICEs likely undergo autonomous rolling circle replication. We propose that they use *oriT* as a *dso*, the conjugative relaxase as a replicative relaxase, and an *sso* for second strand synthesis following conjugative transfer to a new host and during autonomous replication in the original host.

### The location of *sso1* relative to *oriT*

**Fig 8:**
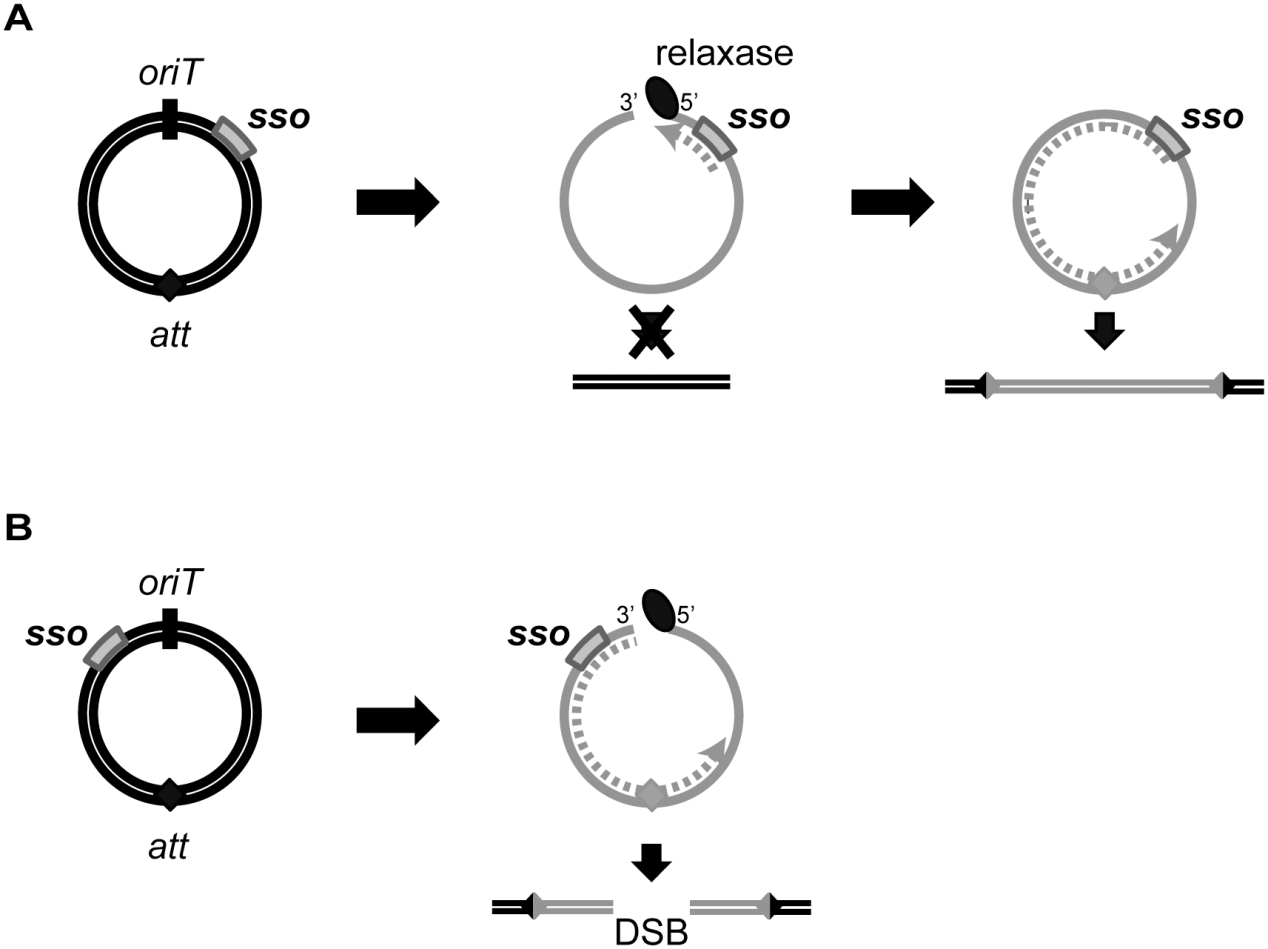
Model of ICE integration with an *sso* downstream (A) and upstream (B) of *oriT*. For all ICEs, excision from the chromosome yields a dsDNA circle (concentric black circles) with an origin of transfer (*oriT*, black slash mark) and the attachment site, *att* (filled diamond). Prior to conjugation, relaxase (black oval) nicks at *oriT* and covalently attaches to the 5’ end. The relaxase, attached to ssDNA (gray solid line) containing the *sso* (gray rectangle), is transferred to recipients. In the transconjugant, the relaxase catalyzes strand ligation and formation of a ssDNA circle [reviewed in 21]. Based on known mechanisms of site-specific recombination, the *att* site must be double-stranded in order for the ICE to integrate into the recipient chromosome (parallel black lines). If the *att* site becomes dsDNA before the nicked DNA is recircularized, and if this incomplete ICE were to integrate into the chromosome, then a double strand break in the chromosome would be created. **(A)** The *sso* in an ICE is downstream from *oriT*. Second strand synthesis (dotted gray line) cannot proceed through *oriT* until the linear DNA becomes circularized. Once the *att* site in ICE becomes double-stranded, site-specific recombination into the chromosome can occur, and the product will be a fully integrated element with an intact genome. **B)** The *sso* in an ICE is upstream from *oriT*. Second strand synthesis (gray dotted line) can occur on the linear DNA, and it is possible that the *att* site becomes double-stranded before the ICE circularizes. If this form of ICE is capable of undergoing site-specific recombination, then integration of this linear dsDNA into the chromosome will yield a double-stranded break (DSB).

The location of *sso1* relative to *oriT* in ICE*Bs1* could increase the probability of successful chromosome re-integration. *sso1* in ICE*Bs1* is downstream of *oriT*, the double-stranded origin of replication (*dso*). In contrast, the *sso* in most RCR plasmids is upstream of the *dso* [23], although there are some exceptions [e.g., 28]. The positioning of an *sso* upstream of the *dso* in plasmids ensures that the *sso* is not single-stranded (and thus active) until leading strand synthesis from the *dso* is almost complete. However, the location of ICE*Bs1 sso1* relative to *oriT* ensures that the attachment site in the circular ICE*Bs1* (*attICE*, previously referred to as *attP*) is not double-stranded (and thus a substrate for site-specific recombination into the chromosome) until ligation and recircularization at the *nic* site (in *oriT*) occurs. Initiation of second strand synthesis from an *sso* upstream of *oriT* could result in replication of *attICE* before recircularization, and integration could result in a double-strand break in the chromosome (Fig 8). Thus, the location of *sso's* in ICEs may be an adaptation to the ICE lifecycle to prevent premature integration and possible damage to the host.

### Mechanisms of second strand synthesis

RCR plasmids and conjugative plasmids have evolved multiple strategies to initiate second strand synthesis [3,24]. The F plasmid of *E*. *coli* and many RCR plasmids from Gram positives contain an *sso* that, when single-stranded, produces a folded structure that is recognized as a promoter by the host RNA polymerase. Transcription initiates and a short RNA serves as a primer for DNA replication [51–55]. Some plasmids (from both Gram negative and positive bacteria) contain sites that recruit the host primase DnaG, and these sites are important for plasmid replication [25,38]. The mobilizable plasmid ColE1 contains a primosome assembly site that is thought to be involved in priming of the transferred strand [3,56]. The *sso* of RCR plasmid pWV01 is RNA polymerase-dependent in *B*. *subtilis* [27] but has RNA polymerase-independent priming activity in *Lactococcus lactis*. This RNA polymerase-independent priming requires a region of the *sso* similar to the primosome assembly site in the phage ø174 [27]. Lastly, several conjugative or mobilizable plasmids from Gram negative bacteria encode a primase that can synthesize RNA primers on the transferred ssDNA [57,58], and primase activity can be important for conjugative transfer [59–61]. The plasmids RSF1010 and R1162 each have a primase that recognizes the plasmid origin of replication (*oriV*) [62,63]. The primases of ColIb and RP4 can prime a variety of ssDNA templates [58] and appear to have general primase activity that is not specific to their cognate plasmid [57,64].

### *sso*-independent second strand synthesis

Despite the loss of regions that contribute to second strand DNA synthesis in ICE*Bs1*, limited or inefficient second strand synthesis most likely occurs. Inefficient second strand synthesis also occurs in many RCR plasmids that are missing an *sso*. Plasmids without an *sso* can be maintained in cells and some are even used as cloning vectors [for example, 37,65]. Although the mechanisms for this *sso*-independent second strand synthesis are not known, one possibility is that RNA fragments that can hybridize to ssDNA could serve as primers for DNA replication. Even though elements that use rolling circle replication can function without an *sso*, any element with an *sso*, and thus an efficient mechanism for priming and completing second strand synthesis, should have a significant evolutionary advantage over elements lacking this function. We suspect that the RCR plasmids and ICEs use similar mechanisms to prime second strand synthesis when there is not an *sso*.

### Possible functions for multiple means of second strand synthesis in ICE*Bs1*

Based on our results, we conclude that ICE*Bs1* has at least two mechanisms for efficient second strand synthesis, one of which utilizes *sso1*. We postulate that these multiple mechanisms might increase propagation of ICE*Bs1* under different conditions in *B*. *subtilis* and perhaps broaden its ability to function in other hosts. For example, there might be growth stages or conditions in which use of one mechanism is inefficient. In this case, the presence of a second mechanism for second strand synthesis could allow for more efficient propagation of ICE*Bs1*.

We also suspect that multiple modes of initiating second strand synthesis could enable ICE*Bs1* to transfer and propagate efficiently to multiple hosts. For example, whereas *sso1* works efficiently in *B*. *subtilis*, it might not work efficiently in another organism, and a different mechanism for initiating second strand synthesis could enable spread and maintenance of ICE*Bs1* in such hosts. Some *sso*'s are known to function in only one or a few host species, and host specificity is determined, in part, by the strength of host-specific RNA polymerase-*sso* interactions and/or the presence of other host factors [23,54]. For example, RCR plasmid pMV158 contains two *sso*'s that are functionally redundant in *Streptoccus pneumoniae*. However, deletion of one of the *sso*'s, *ssoU*, decreases conjugative transfer of pMV158 from *S*. *pneumoniae* to *Enterrococcus faecalis* by 300-1000-fold [28,66]. The conjugative module from pMV158 composed of its cognate MobM relaxase, *oriT* and two *sso*’s is widely conserved in plasmids from several Gram positive species [66]. Similarly, the primases of conjugative plasmids ColIb and RP4 are important for conjugation into some species but disposable for conjugation into others, indicating that second strand synthesis mechanisms differ between hosts [3]. We speculate that many ICEs might have two different regions that allow for conversion of ssDNA to dsDNA, perhaps enabling stable acquisition by and maintenance in different host species and thereby broadening the ICE host range.

## Materials and Methods

### Strains and alleles

*B*. *subtilis* strains were derived from JH642 (*pheA1 trpC2*) [67,68] and are listed in Table 1. Most were constructed by natural transformation. Conjugation experiments utilized recipient CAL85 that is cured of ICE*Bs1* (ICE*Bs1* ^0^) and is resistant to streptomycin (*str-84*) [5]. To induce ICE*Bs1* gene expression in host cells, *rapI* was overexpressed from *amyE*::{(Pxyl-*rapI*) *spc*} [9] or *amyE*::{(Pxyl-*rapI*) *cat*} [43]. Pxyl is a xylose-inducible promoter that is also repressed in the presence of glucose [69]. Construction of the non-excisable ICE*Bs1* in *thrC325*::{(ICE*Bs1-311* Δ*attR*::*tet*) *mls*}) has been described previously [7].

Most ICE*Bs1* derivatives contained *kan*, conferring resistance to kanamycin. The Δ(*rapI-phrI*)*342*::*kan* allele in LDW21 and LDW22 has been described [5]. The Δ(*conG-yddM*)*39*::*kan* and Δ(*yddB-yddM*)*41*::*kan* mutations have the same endpoints as alleles described previously [7]. Plasmids pCAL316 and pCAL317 containing *kan* and ~1 kb of DNA flanking the deletion were linearized and transformed into the ICE*Bs1* markerless Δ*sso1* mutant (Δ*sso1-13*) to generate ICE*Bs1* Δ*sso1-13* with Δ(*conG-yddM*)39::*kan* and Δ(*yddB-yddM*)41::*kan*, respectively. PCR was used to confirm the mutations in ICE*Bs1* and verify that they were produced by double crossover recombination.

Δ*sso1-13* is a 194-nucleotide markerless deletion that fuses the 43^rd^ and 238^th^ nucleotides of the intergenic region between *nicK* and *ydcS*. Two 1.5 kb DNA fragments containing DNA flanking the deletion site were PCR amplified. The two fragments were fused and inserted into the BamHI and EcoRI sites of pCAL1422 (a plasmid that contains *E*. *coli lacZ*) [8] via isothermal assembly [70]. The isothermal assembly product was integrated by single crossover into *B*. *subtilis* strain IRN342 (ICE*Bs1* Δ(*rapI-phrI*)*342*::*kan*) [5]. Transformants were screened for loss of *lacZ*, indicating loss of the integrated plasmid, and PCR was used to identify a Δ*sso1* (LDW22) and wild type (LDW21) clone.

Mini-ICE*Bs1 sso1*+ {ΔICE*Bs1-93* Δ(*ydcS-yddM*)*93*::*kan*} and mini-ICE*Bs1* Δ*sso1* {ΔICE*Bs1-177* Δ(*sso1-yddM*)*177*::*kan*} are large deletion-insertions that leave the left and right ends of ICE*Bs1* intact. Both deletions contain a kanamycin resistance gene that interrupts part of *yddM* as previously described [6]. The Δ(*ydcS-yddM*)*93*::*kan* deletion-insertion begins 19 bp upstream of *ydcS*. The Δ(*sso1-yddM*)*177*::*kan* deletion-insertion begins 6 bp downstream of *nicK*. Splice-overlap-extension PCR [71] was used to fuse a ~1 kb fragment of genomic DNA upstream of the deletion endpoint to a DNA fragment containing *kan* and the *kan-yddM* junction amplified from ΔICE*Bs1-205* [6].

We constructed two pHP13 derivatives to test *sso1* function. Both plasmids contain *sso1* (based on conservation to the Sso of plasmid pTA1060) and additional flanking sequence from ICE*Bs1*. We used PCR to amplify a 418 bp fragment of ICE*Bs1* from 78 bp upstream of the 3' end of *nicK* to the first 76 bp of *ydcS*, including *sso1* (Fig 1). The PCR product was ligated into the *lacZ* alpha complementation region of pHP13 [37,39] (NCBI accession DQ297764.1) with the multiple cloning sites, between the BamHI and EcoRI sites (pCJ44) or SalI and EcoRI sites (pCJ45). pCJ44 contains *sso1* in a functional orientation and pCJ45 contains *sso1* in the reverse orientation (sso1R), relative to the direction of leading strand DNA synthesis [37].

The *ssb-mgfpmut2* fusion is driven by the *rpsF* promoter and inserted by double crossover at *lacA*, as described previously [40]. Strains with this fusion also contain wild type *ssb* at the normal chromosomal location.

### Media and growth conditions

*Bacillus subtilis* cells were grown in LB or in MOPs-buffered S7_50_ defined minimal medium [72]. ICE*Bs1*-containing strains were grown in minimal medium containing arabinose (1% w/v) as the carbon source, and ICE*Bs1* gene expression was induced by the addition of xylose (1% w/v) to induce expression of Pxyl-*rapI*. Cells containing pHP13-derived plasmids were grown in liquid medium containing 2.5 μg/ml chloramphenicol to select for maintenance of the plasmid. Chloramphenicol was omitted from the growth medium when testing for maintenance of pHP13-derived plasmids as described in the text. Antibiotics were otherwise used at the following concentrations: kanamycin (5 μg/ml), chloramphenicol (5 μg/ml), spectinomycin (100 μg/ml), tetracycline (10 μg/ml), streptomycin (100 μg/ml), and a combination of erythoromycin (0.5 μg/ml) and lincomycin (12.5 μg/ml) to select for macrolide-lincosamide-streptogramin (*mls*) resistance.

### Conjugation assays

Conjugation experiments were performed essentially as described [5,6]. Briefly, donor and recipient cells were grown in defined minimum medium containing 1% arabinose. Xylose (1%) was added to donors to induce expression of Pxyl-*rapI*, causing induction of ICE*Bs1* gene expression and excision. After two hours of growth in the presence of xylose, equal numbers of donor and recipient cells were mixed and collected by vacuum filtration on a nitrocellulose filter. Filters were incubated at 37°C for 3 hours on 1.5% agar plates containing 1X Spizizen’s salts (2 g/l (NH_4_)SO_4_, 14 g/l K_2_HPO_4_, 6 g/l KH_2_PO_4_, 1 g/l Na_3_ citrate-2H_2_O, 0.2 g/l MgSO_4_-7H_2_0) [73]. Cells were washed from the filters, diluted and plated on LB agar containing streptomycin and kanamycin to select for transconjugants. Donor cell concentration was determined at the time of cell mixing (after growth in xylose for two hours) by plating donor cells on LB agar containing kanamycin. Conjugation efficiency was calculated as the ratio of transconjugants per donor.

### Live cell fluorescence microscopy

Microscopy was performed essentially as described [74,75]. Briefly, mid-exponential phase cells were placed on pads of 1% agarose. Images were taken on a Nikon E800 microscope equipped with Hamatsu CCD camera and 100X DIC objective. Chroma filter set 41012 was used for GFP. The contrast and brightness of fluorescent images were initially processed using Improvision Openlabs 4.0 Software.

We measured the intensity and area of Ssb-GFP foci using ImageJ (http://imagej.nih.gov/ij/). A high (conservative) global threshold was applied to every image to separate intense Ssb-GFP foci (pixel intensity ≥ 200, 8-bit image) from background. The area of each intense Ssb-GFP focus was measured using the automatic particle analysis tool. We then analyzed the distribution of the area of each focus, and used 4 pixels as a cutoff for a “large” focus (4 pixels was the third quartile for intense foci in control strain CMJ118). Finally, we counted the number of cells in the DIC image, and calculated the number of cells with at least one large, intense focus.

### Southern blots

Mid-exponential phase cultures were fixed in an equal volume of ice-cold methanol. Cells were washed in NE buffer (100 mM NaCl, 50 mM EDTA, pH 8.0) and lysed in NE buffer containing 0.5 mg/ml lysozyme for 30 min at 37°C. Sarcosyl (1% final, Sigma) and proteinase K (90 μg/ml final, Qiagen) were added, and the suspension was incubated for 20 min at 70°C. Equal volumes of phenol and chloroform were added, and the suspension was vortexed vigorously. Following centrifugation, the aqueous layer was removed and total nucleic acids were precipitated from the aqueous layer by addition of 0.1 volume of 3 M sodium acetate and 2.5 volumes of ice-cold ethanol. The precipitate was washed once with 70% ethanol and resuspended in ddH_2_O overnight at room temperature.

Equal amounts of nucleic acid (~40 μg per sample) were separated on two 0.8% agarose gels. Following electrophoresis, one of the gels was soaked in an alkaline solution (0.5 M NaOH, 1.5 M NaCl) for 30 min to denature the DNA. Both gels were also soaked in neutralization buffer (2.5 M NaCl, 0.5 M Tris HCl) for 30 min. DNA was transferred to nicrocellulose membranes (Whatman) by capillary transfer, essentially as described [76]. DNA was then fixed by baking the membranes for 2 hours at 80°C. Prior to probing, the membranes were incubated for 1 h at 37°C in rotating tubes containing prehybridzation buffer with formamide [76].

We used ^32^P-labeled probe to detect plasmid DNA. Primer CLO377 (5’-AGCACCCATT AGTTCAACAA ACG-3’, complementary to part of *cat* on pHP13) was end-labeled with (gamma-^32^P)-ATP (Perkin-Elmer) using T4 polynucleotide kinase (New England Biolabs). Labeled oligonucleotides were separated from unincorporated ATP using Centri-Spin 10 spin columns (Princeton Separations). A region of *cat* from pHP13 was PCR amplified using labeled CLO377 and unlabeled primer oLW39 (5’-AGTCATTAGG CCTATCTGAC AATTCC-3’), thereby producing dsDNA with one strand labeled. The PCR product was separated from excess primers using the Qiagen PCR Purification Kit and diluted in hybridization buffer [76]. The PCR product was denatured by boiling for 1 min and immediately put on ice. Equal amounts of probe were applied to each blot (denatured and non-denatured). Membranes and probe were incubated overnight at 37°C in rotating tubes containing hybridization buffer. Excess probe was removed from the membranes by serially washing in 2X—0.1X SSC and 0.5%- 0.1% SDS. The ^32^P-labeled DNA was detected using a Typhoon FLA 9500 phosphorimager.

### Chromatin immunoprecipitation

ChIP-qPCR was used to measure the association of Ssb-GFP with pHP13-derived plasmid DNA and was carried out essentially as described [77,78]. Briefly, DNA-protein complexes were crosslinked with formaldehyde. Ssb-GFP was immunoprecipitated with rabbit polyclonal anti-GFP antibodies (Covance). qPCR was used to determine the relative amount of plasmid DNA that was bound to Ssb-GFP [42]. We used primers specific to the *cat* gene in the pHP13 backbone to amplify DNA in both immunoprecipitates and in pre-immunoprecipitation lysates (representing the total input DNA). Values from immunoprecipitates were normalized to those of total input DNA (% of input) [42].

We also determined the amount of total plasmid in each strain relative to control strain CMJ129 using the ΔΔ*Cp* method [79]. DNA was amplified from pre-immunoprecipitated lysates, and values obtained for plasmid gene *cat* were normalized to chromosomal locus *ydbT*. Primers to *cat* were oLW104 (5’-GCGACGGAGA GTTAGGTTAT TGG-3’) and oLW107 (5’-TTGAAGTCAT TCTTTACAGG AGTCC-3’). Primers to *ydbT* were described [8].

### Integration of ICE*Bs1*

We used qPCR to determine if ICE*Bs1* was integrated into the chromosomes of transconjugants. We measured *attL*, the junction between the chromosome and the left end of ICE*Bs1* and *attB*, the chromosomal attachment site without ICE*Bs1* using primer pairs specific for each region [5,30].

We also used qPCR to measure reintegration of ICE*Bs1* into the chromosome of cells from which it originally excised. In these experiments, host cells with ICE*Bs1* integrated in the chromosome at *attB* were grown in defined minimal medium with 1% arabinose. Expression of Pxyl-*rapI* was induced with 1% xylose, causing induction of ICE*Bs1* gene expression and excision. After two hours of growth, cells were pelleted and resuspended (to an OD_600_ of 0.05) in minimal medium with 1% glucose (and no xylose) to repress expression of Pxyl-*rapI* and eventually restore repression of ICE*Bs1* gene expression. DNA was extracted at various times after repression of Pxyl-*rapI* from 1–2 ml of cell culture using the Qiagen DNEasy tissue kit protocol for Gram-positive bacteria.

We determined the amount of reintegrated ICE*Bs1* relative to uninduced cells (before expression of Pxyl-*rapI*) using the ΔΔ*Cp* method [79]. ICE*Bs1* reintegration was determined by quantifying the amount of *attL*, the junction between the chromosome and the left end of ICE*Bs1* [30], relative to the amount of the nearby chromosomal locus *ydbT*. Values were normalized to uninduced cells in which ICE*Bs1* is integrated in a single copy in the chromosome.
